# Evaluation of antimicrobial, antioxidant and cytotoxic properties of bioactive compounds produced from endophytic fungi of Himalayan yew ( *Taxus wallichiana*) in Nepal

**DOI:** 10.12688/f1000research.23250.2

**Published:** 2020-10-05

**Authors:** Dhurva Prasad Gauchan, Pratistha Kandel, Astha Tuladhar, Ashesh Acharya, Upendra Kadel, Aayush Baral, Arjan Bir Shahi, María Rosario García-Gil

**Affiliations:** 1 Department of Biotechnology, School of Science, Kathmandu University, Dhulikhel, Kavrepalanchok District, Bagmati, 45200, Nepal; 2 Department of Forest Genetics and Plant Physiology, Umeå Plant Science Centre, Swedish University of Agricultural Sciences, Umeå, SE-901 83, Sweden

**Keywords:** Taxus wallichiana, Endophytic, DPPH, GAE, RE, cytotoxic

## Abstract

**Background:** Endophytic fungi are largely underexplored in the discovery of natural bioactive products though being rich sources of novel compounds with promising pharmaceutical potential. In this study, *Taxus wallichiana,* which has huge medicinal value, was investigated for its endophytic diversity and capability to produce bioactive secondary metabolites by analyzing antioxidant, antimicrobial and cytotoxic properties.

**Methods: **The endophytes were identified by ITS-PCR using genomic DNA samples. The secondary metabolites were extracted by solvent extraction method using ethyl acetate. The antioxidant activity was analyzed by Thin Layer Chromatography, Total Phenol Content (TPC), Total Flavonoid Content (TFC) and DPPH assay, and the antimicrobial activity was analyzed by agar-well diffusion method. Brine shrimp lethality assay was used to analyze the cytotoxicity of the fungal extracts.

**Results:** Out of 16 different *Taxus* trees sampled from different locations of Dhorpatan, 13 distinctive endophytic fungi were isolated and grouped into 9 different genera: *Bjerkandera, Trichoderma, Preussia, Botrytis, Arthrinium, Alternaria, Cladosporium, Sporormiella *and *Daldinia*. The ethyl acetate extracts isolated from three endophytic fungi: *Alternaria alternata*, *Cladosporium cladosporioides* and *Alternaria brassicae* showed significant TPC values of 204±6.144, 312.3±2.147 and 152.7±4.958µg GAE/mg of dry extract, respectively, and TFC values of 177.9±2.911, 644.1±4.202 and 96.38±3.851µg RE/mg of dry extract, respectively. Furthermore, these three extracts showed a dose dependent radical scavenging activity with IC _50_ concentration of 22.85, 22.15 and 23.001 µg/ml, respectively. The extracts of *C. cladosporioides* and *A. brassicae* also showed promising antimicrobial activity against *Escherichia coli*, *Staphylococcus aureus* and *Bacillus subtilis* with a minimum inhibitory concentration of 250μg/ml for all bacteria. Both the samples showed cytotoxic property against shrimp nauplii with LC _50_ of 104.2 and 125.9µg/ml, respectively.

**Conclusions:** The crude fungal extracts obtained from endophytes: *A. alternata*, *C. cladosporioides* and *A. brassicae *upon purification and further identification of the bioactive compounds can be a fascinating source for novel pharmaceutical agents.

## Introduction

*Taxus wallichiana* Zucc. (Himalayan yew) is an evergreen coniferous tree (10 to 28 meters in height), native to the Himalaya and parts of southeast Asia that grows on steep, moist mountain slopes at altitudes of about 2000 m to 3500 m above sea level. It has flat and dark green leaves, which are arranged spirally on the stem ^1^. The leaves and bark of *Taxus* species are the primary source of the chemotherapeutic drug Paclitaxel used in the treatment of breast and ovarian cancer, resulting in it receiving huge attention worldwide ^2^. The Himalayan yew also has an exceptional history of its application in the traditional system of medicine for the treatment of fever and painful inflammatory conditions. It is also consumed as decoctions, herbal tea, and juice for treating cold, cough, respiratory infections, indigestion, and epilepsy and also on infected wounds and burns as poultice ^1, 3^. Some research indicates various medicinal properties of *T. wallichiana,* such as analgesic, antipyretic, anti-inflammatory, immunomodulatory, antibacterial, antifungal, antiplatelet, antispasmodic, antiallergic, anticonvulsant, antiosteoporotic, and vasorelaxing effect ^3– 6^. Due to the persistent over-exploitation of Himalayan yew for its leaves and bark, it is now on the verge of extinction and classified as endangered by the International Union for Conservation of Nature (IUCN). Alternative measures to utilize the medicinal components of *Taxus* while also addressing the vexing problem of its mass exploitation are needed.

Plants growing in unique environmental conditions or strange locations and having ethnobotanical uses generally harbour novel endophytic microorganisms ^7^. Endophytes are microbial species that colonize the inner tissues of higher plants at some time in their life cycles without causing apparent damage or producing external structures ^8, 9^. There is a symbiotic interaction between endophytes and their host plants that may result in mutualistic benefits for both partners, such that these endophytes may provide protection and survival conditions for the plants by producing a myriad of bioactive substances and in return obtain carbon for energy. These bioactive substances exhibit important properties, such as antioxidant, anticancer, antimicrobial, cytotoxic, immunomodulatory, antiviral, anti-parasitic and insecticidal properties, which when isolated and characterized, may also have the potential for use in industry, agriculture, and medicine ^10– 12^. The majority of endophytic fungi possess biosynthetic capabilities similar to the associated host because of their long history of co-evolution and genetic recombination.

The development of resistance by pathogenic microorganisms to available drugs is a growing problem, which leads to an immediate need for an intensive search for newer and effective antimicrobial agents. Many endophytic fungi have the ability to produce antimicrobial substances ^13^. Our body continuously produces reactive oxygen species (ROS) as by-products of aerobic metabolism ^14^. Excessive production of these ROS can damage our cells, tissues and organs and can inhibit the normal function of DNA. These damages can in turn cause various diseases such as cancer, male factor infertility, heart disease and Alzheimer’s disease ^14– 16^. Fungi can potentially produce a wide variety of antioxidants such as alkaloids, flavonoids, phenols, steroids, etc. that can scavenge the excessive ROS produced in our body ^17^.

Though sufficient research has shown that endophytic fungi harboring *T. wallichiana* have the capability to produce Taxol, to our knowledge, no reports are available on antioxidant, antimicrobial and cytotoxic properties of these endophytes. This study focuses on investigating *T. wallichiana* for its endophytic fungal diversity and capability of these fungal endophytes to produce bioactive secondary metabolites having antimicrobial, antioxidant and cytotoxic properties. This study aims to establish endophytic fungi as sustainable natural reservoirs for novel drug discovery useful in medicine, food and agriculture industries.

## Methods

### Sample collection

**Figure 1.**
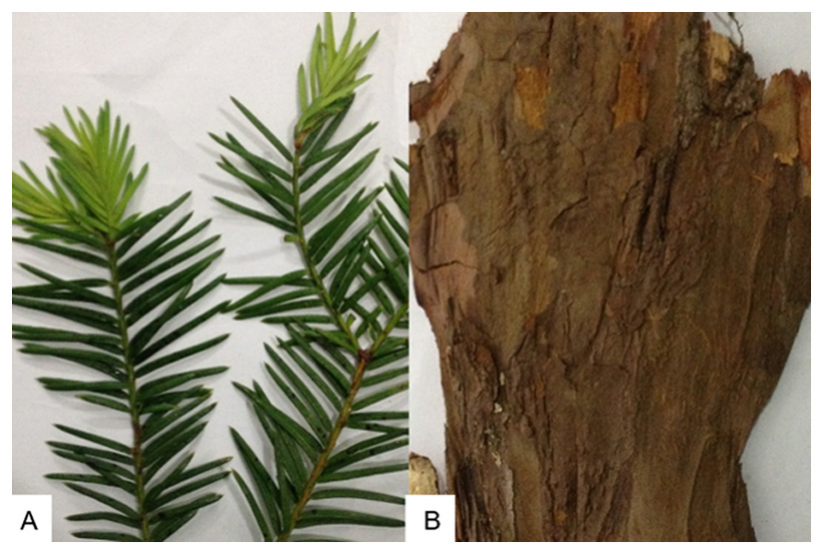
*Taxus wallichiana* plant sample collected from the periphery of Dhorpatan Hunting Reserve. ( **A**) Twigs with leaf needles of *T. wallichiana.* ( **B**) Bark of *T. wallichiana*.

Bark and twigs with leaf needles of 16 different *T. wallichiana* trees were collected from the periphery of Dhorpatan Hunting Reserve using the simple random sampling method in the month of May, 2018. Dhorpatan is a village in Nepal’s Baglung District at 2900 m elevation. The collected samples were placed in sterile polyethylene bags. The samples were first verified with consultation of the local people under the vernacular name *‘Lauth Salla’* and then from the District Forest Office, Baglung. Bark and leaf samples collected are shown in Figure 1.

### Isolation of endophytic fungi

Bark and needles were cut and washed thoroughly under running tap water to remove the dirt and debris. The pieces of bark and leaves were treated with 70% ethanol (v/v) for 1 minute and 0.1% mercuric chloride (v/v) for 2 minutes and rinsed twice with sterile distilled water. For bark, the outer layer was removed by a sharp, sterilized blade to expose the inner layer. The inner pinkish-white layer was placed on the surface of potato dextrose agar (PDA) media. For needles, the individual needle was cut from the midrib and also from edges by a sterile sharp blade and placed on the surface of PDA media exposing the inner tissues. In another method, both bark and needles were ground into a paste with 2 ml of sterilized water using mortar and pestle separately under aseptic conditions. 1ml of bark paste and 1 ml of paste was poured on Petri plates and molten PDA media was poured into each plate. PDA plates were then incubated at 25±2°C for 10 days to allow the growth of endophytic fungi. Pure culture of fungal endophytic isolates was obtained by hyphal-tip transfer to fresh PDA media.

### Characterization of isolated endophytic fungi

***Morphological screening***. Microscopic characterization of the isolated endophytic fungi was carried out using the tape lift mount method using lactophenol cotton blue as dye and observed under 40X, 100X and 400X magnification in a compound microscope and then photographs were captured by a digital camera. Shape, color, margin, elevation, spore structure and other morphological characteristics of the endophytic isolates were observed.

***Extraction of secondary metabolites***. Pieces of agar blocks (10 mm) of the mycelial mats from an actively growing fungal culture was inoculated into 250 ml Erlenmeyer flasks containing 100 ml potato dextrose broth media using a sterile borer. Flasks were then incubated at 150 rpm at 25±2°C for 10 days in a shaking incubator. The entire fermented broth was then filtered through filter paper. An equal volume of ethyl acetate was added to the filtrate broth and vigorously shaken for 5 minutes. The mixture was transferred to a separating funnel and the organic layer of ethyl acetate was allowed to separate from the aqueous layer. The ethyl acetate fraction, which comprised of the upper organic layer in the separating funnel, was then transferred to a new flask and allowed to dry at room temperature using an evaporator. The dried extract obtained from each fungal sample was weighed and stored at 4°C for future use. The percent yield of fungal extracts were calculated using the formula: Percent yield = weight of crude extract (mg)/ Total weight of fungal powder (mg) * 100 ^18^.

***Molecular screening and phylogenetic analysis***. Genomic DNA of endophytic fungi was isolated using Quick-DNA™ Fungal/Bacterial Miniprep Kit from Zymo Research, as per the manufacturer’s instructions. The obtained genomic DNA was analyzed using 0.8% agarose gel electrophoresis and the bands were observed. The isolated DNA was used as a template to amplify the ITS regions together with the 5.8s rRNA gene (ITS 1-5.8S-ITS2 rDNA) through PCR using universal primers ITS1 (5'TCCGTAGGTGAACCTGCGG-3') and ITS4 (5'-TCCTCCGCTTATTGATATGC-3'). PCR was performed using all reagents from Thermofisher, in a 25 µl reaction mixture using 5 µl template DNA, 0.25 µl of 1.25 U Taq DNA polymerase, 2.5 µl of 10X Amplification buffer, 0.8 µl of 10mM dNTP, 0.75 µl of 50 mM MgCl _2_, 13.2 µl PCR grade water, 1.25 µl of 10 μM each of forward and reverse primer. PCR amplification was carried out in BioRad300 thermocycler. The thermo-cycling conditions involved an initial denaturation at 94°C for 3 mins, 37 cycles of 30 secs at 94°C, 30 secs at 55°C, 1 min at 72°C and a final extension at 72°C for 5 mins. The PCR amplification products were analyzed by gel electrophoresis on 2% agarose (w/v). 100 bp DNA ladder from Thermo-fisher was used. The remaining volume was sent for sequencing to Macrogen, Korea.

The ITS sequences of fungal isolates were subjected to BLASTn sequence homology search and compared with data in the National Centre for Biotechnology Information (NCBI). Multiple sequence alignment of related species was performed using CLUSTAL W. Phylogenetic analysis was carried out by Maximum Likelihood (ML) method based on the Tamura-Nei model ^19^ using MEGA X software. Initial tree(s) for the heuristic search were obtained by Neighbor-Join and BioNJ algorithms to a matrix of pairwise distances estimated using the Maximum Composite Likelihood (MCL) approach, and then selecting the topology with superior log likelihood value ^20^. One thousand bootstrap replicates were analyzed to assess reliable nodal support values. The colonization frequency (%CF) of endophytic fungi was calculated using the formula: %CF = (Number of segments colonized by a single endophyte / Total number of segments observed) * 100

### Qualitative analysis of antioxidant activity using thin layer chromatography (TLC)

Benzene: Chloroform: Acetone: Methanol (20:92.5:15:7.5) was used as the solvent system. The sample was prepared by dissolving the fungal extracts in ethyl acetate. TLC was carried out on 20×20 alumina TLC plate. Ascorbic acid, Rutin, Quercetin, and Gallic acid were used as standards. The samples were loaded equidistantly onto the TLC plate and allowed to air dry. The TLC plate was allowed to run in the TLC chamber containing the mobile phase. The TLC plate was then air-dried and sprayed with 10% AlCl _3_ solution followed by 0.04% DPPH solution.

### Quantitative analysis of antioxidant activity

***DPPH radical scavenging assay***. DPPH radical scavenging assay was performed as proposed by Makris *et al.* with minor modifications ^21^. DPPH reagent (0.04 mg/ml) was prepared in methanol. Ascorbic acid (Vitamin C) was used as a positive control. Different concentration of ascorbic acid as well as fungal extracts were made (2, 4, 6, 8, 10, 12, 14, 16, and 18 µg/ml) from 1 mg/ml stock solution. These samples were mixed with DPPH reagent in the ratio 1:1 and incubated at 27°C in the dark for 30 minutes. The radical scavenging activity of the samples against DPPH free radical was determined by measuring the UV absorbance at 517 nm against the blank. The mixture of 1 ml DPPH solution and 1 ml blank methanol was used as control. The radical scavenging activity of the standard (Ascorbic acid), as well as the samples (fungal extracts), were calculated using the formula:

DPPH radical scavenging activity (%) = (Abs control – Abs sample)/Abs control × 100

Where Abs control is the absorbance of control and Abs sample is absorbance of test samples. From calibration curves obtained from different concentrations of the extracts and ascorbic acid, the concentration of sample required to scavenge 50% of DPPH free radicals (50% inhibition concentration, IC _50_) was determined.

***Total phenol content (TPC)***. The TPC of the fungal endophytes was determined using the Folin-Ciocalteu method described by Lin and Tang with minor modifications ^22^. Each extract solution (0.5 mg/ml, 1 ml) was diluted to 5 ml deionized water in a 25 ml test tube. 0.25 ml volume of Folin-Ciocalteu reagent and 5 ml of 7.5% Sodium carbonate (Na _2_CO _3_, w/v) were added. This mixture was incubated at 40°C for 60 mins, diluted with deionized water up to 12.5 ml total volume and mixed. The absorbance was measured at 733 nm against the blank on a spectrophotometer. The TPC was calculated on the basis of the Gallic acid calibration curve. The results were expressed as μg of Gallic acid equivalents (GAEs) per mg of dry extract.

***Total flavonoid content (TFC)***. The TFC was determined using NaNO _2 ^-^_Al(NO _3_) _3 ^-^_NaOH colorimetric assay described by Zhu *et al.* with minor modifications ^23^. Each extract solution (0.5 mg/ml, 1 ml) and 2 ml of 60% alcoholic solution (v/v) were accurately added to a 25 ml test tube and 0.5 ml of NaNO _2_ solution (5% w/v) was added. The mixture was shaken and left to stand for 6 mins. 0.5 ml of the Al(NO _3_) _3_ (10% w/v) solution was added. After 6 mins, 5 ml of NaOH solution (10% w/v) was added, followed by dilution with deionized water up to 12.5 ml volume. The solution was mixed and incubated at room temperature for 15 mins. The reaction mixture absorbance was measured at 507 nm against the blank in the spectrophotometer. The TFC was calculated on the basis of the calibration curve of Rutin. The results were expressed as μg of Rutin Equivalents (RAEs) per mg of dry extract.

### Antimicrobial activity assay

***Agar-well diffusion method***. The antimicrobial activity of the fungal extracts was determined using the agar-well diffusion method. A gram-negative bacterial strain: *Escherichia coli* (ATCC 25922), and two gram-positive bacterial strains: *Staphylococcus aureus* (ATCC 12600) and *Bacillus subtilis* (ATCC6633) were used as indicator organisms. The dried extracts of each fungal isolate were dissolved in dimethyl-sulphoxide (DMSO). The microbial cultures were spread evenly using L-shaped spreader. Wells of 6 mm diameter were made on the plates using a sterile borer. 30 µl of 7 mg/ml of fungal extract dissolved in DMSO was added to the well. DMSO was used as negative control and Gentamycin (10μg disc) was used as a positive control. The plates were incubated at 37±2°C overnight. The zones of inhibition were observed and measured.

***Determination of minimum inhibitory concentration (MIC)***. 2 mg/ml stock solution of fungal extract was diluted into concentrations of 1000, 500, 250, 125, 62.5, 31.25, 15.625, 31.25, 15.625, 7.81, 3.9 and 1.95 μg/ml. Each concentration of extract was mixed with Muller Hinton Broth (MHB) in equal volume to make a total volume of 500 µl. 500 μl of *E. coli* culture was added to the test tubes to make a final volume of 1 ml. Kanamycin sulphate was also diluted into concentrations of 1000, 500, 250, 125, 62.5, 31.25, 15.625, 31.25, 15.625, 7.81, 3.9 and 1.95 μg/ml and used as positive control. Each concentration of kanamycin sulphate was mixed with MHB in equal volume to make a total volume of 500 µl. 500 μl of *E. coli* culture was added to the test tubes to make a final volume of 1 ml. Control 11, which consisted of media, DMSO and *E. coli* culture, was taken as negative control and Control 12, which consisted of media and DMSO only, was taken to test the sterility of the process. All test tubes were incubated at 37°C overnight. The MIC of fungal extracts were determined by observing the turbidity and clarity of the test tubes.

***Cytotoxicity assay by using brine shrimp***. Brine shrimps ( *Artemia salina*) were hatched using brine shrimp eggs in a vessel filled with simulated sterile artificial seawater (brine solution). 38 g of sea salt, sodium chloride was dissolved in 1000 mL of distilled water and the pH was adjusted to 8.5 using 1N NaOH under constant aeration for 48 hours. The active shrimps were collected and used for the assay ^24^. Samples were prepared by dissolving crude fungal extracts in 1% aqueous DMSO to give stock solutions. From the stock, five different concentrations: 1,000, 500, 250, 125, and 62.5 ppm were prepared in seawater. An aliquot of each concentration (0.5 ml) was transferred in triplicate into sterile vials, and aerated seawater (4.5 ml) was added. Ten shrimp nauplii were transferred to each vial. Doxorubicin 1% aqueous solution and 1% DMSO in seawater were used as positive and negative controls, respectively. After 24 hrs, the numbers of live nauplii were counted and the percentage of death was calculated. The concentration that killed 50% of the nauplii (LC _50_ in μg/mL) was determined. Criterion of toxicity for fractions was established i.e. LC _50_ values > 1000 μg/ml (non-toxic), ≥ 500 and ≤ 1000 μg/ml (low toxicity), and < 500 μg/ml (toxic) ^25^.

## Results and discussion

### Isolation of endophytic fungi

Bark and leaf samples from 16 different trees of *T. wallichiana* were collected. The information regarding the names of plant samples, geographical features of the location and the girth of the tree from which samples were collected are given in Table 1.

**Table 1.** Sample code, location and girth of 16 different *Taxus wallichiana* trees sampled from Dhorpatan, Nepal.

S.N.	Tree code	Altitude (m)	Latitude	Longitude	Girth of tree (cm)	Reproductive morphology
1.	S-1	2851	28⁰28.402' N	083⁰02.363' E	46	F
2.	S-2	2861	28⁰28.404' N	083⁰02.352' E	79	M
3.	S-3	2875	28⁰28.401' N	083⁰02.346' E	25	F
4.	S-4	2893	28⁰28.413' N	083⁰02.322' E	82	F
5.	S-5	2857	28⁰28.380' N	083⁰02.375' E	72	F
6.	S-6	2861	28⁰28.389' N	083⁰02.325' E	41	F
7.	S-7	2876	28⁰28.383' N	083⁰02.326' E	44	F
8.	S-8	2875	28⁰28.415 N	083⁰02.310' E	23	F
9.	S-9	2879	28⁰28.422' N	083⁰02.308' E	27	M
10.	S-10	2848	28⁰28.340' N	083⁰02.374' E	42	M
11.	S-11	2847	28⁰28.328' N	083⁰02.372' E	34	F
12.	S-12	2846	28⁰28.371' N	083⁰02.375' E	62	F
13.	S-13	2845	28⁰28.328' N	083⁰02.371' E	55	M
14.	S-14	2845	28⁰28.323' N	083⁰02.377' E	58	M
15.	S-15	2844	28⁰28.325' N	083⁰02.377' E	57	F
16.	S-16	2841	28⁰28.329' N	083⁰02.387' E	51	F

**Figure 2.**
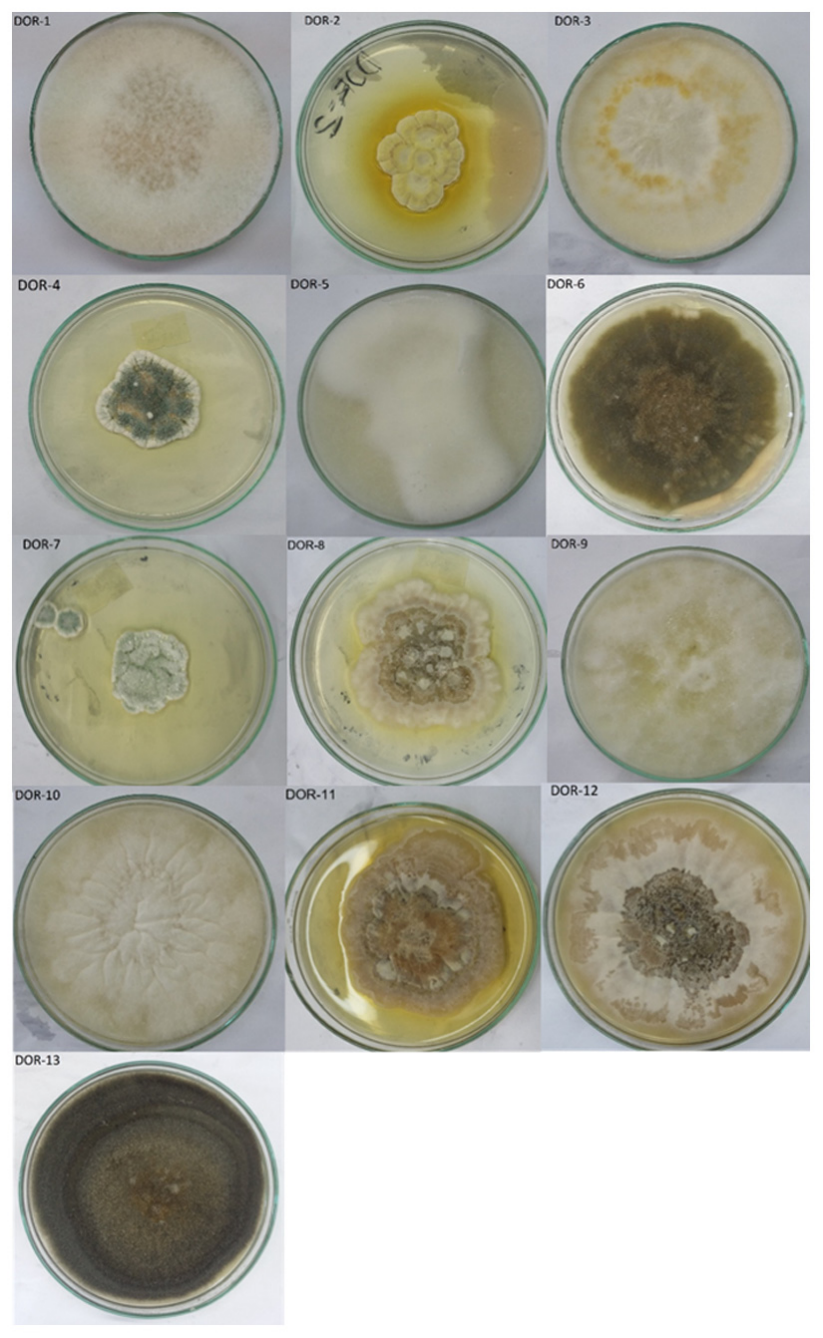
Pure endophytic fungal isolates. Pure culture of 13 fungal endophytic isolates (DOR-1 to DOR-13) obtained from bark and leaves of *Taxus wallichiana* by hyphal-tip transfer to fresh potato dextrose agar media.

**Figure 3.**
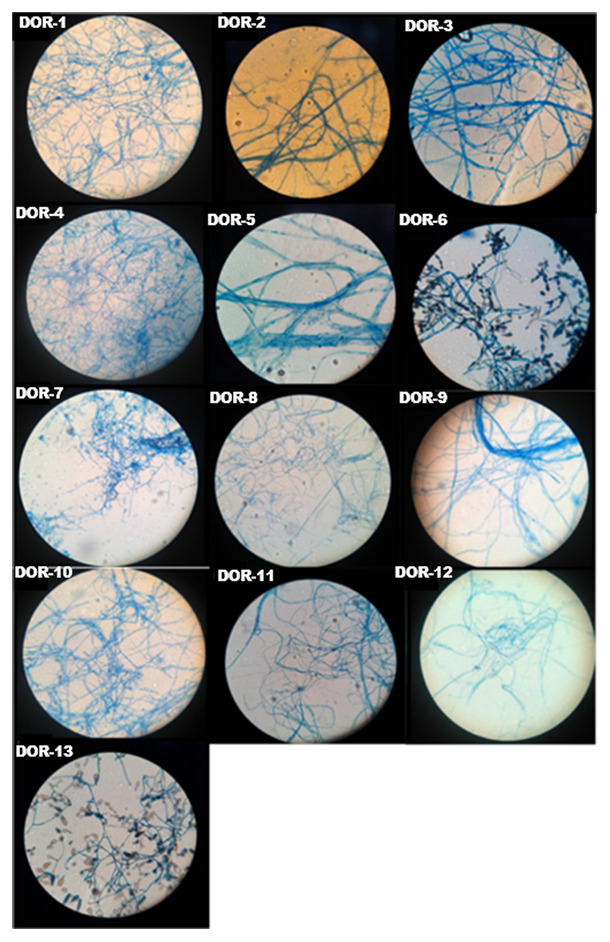
Microscopic view of all fungal isolates. Microscopic characterization of the isolated endophytic fungi was carried out using tape lift mount method using lactophenol cotton blue and viewed under 400x magnification in a compound microscope.

Out of the 16 bark and leaf samples collected, 13 endophytic fungal isolates with different morphological features were obtained. The name codes given to the individual plant samples and their respective fungal isolates are shown in Table 2. The pure fugal isolates obtained are shown in Figure 2 and the microscopic view of all the fungal isolates are shown in Figure 3.

**Table 2.** Details of 13 endophytic fungi with different morphological characters isolated from different plant samples.

S.N.	Sample code	Type of plant material	Fungal isolates codes
1.	S-5-GB	Ground bark	DOR-1
2.	S-15-GB	Ground bark	DOR-2
3.	S-11-IB	Intact bark	D0R-3
4.	S-3-GB	Ground bark	DOR-4
5.	S-2-GB	Ground bark	DOR-5
6.	S-5-GL	Ground leaves	DOR-6
7.	S-3-GB	Ground bark	DOR-7
8.	S-16-GL	Ground leaves	DOR-8
9.	S-11-GL	Ground leaves	DOR-9
10.	S-7-GB	Ground bark	DOR-10
11.	S-15-GL	Ground leaves	DOR-11
12.	S-16-GL	Ground leaves	DOR-12
13.	S-9-GL	Ground leaves	DOR-13

GB: Ground Bark; IB: Intact Bark; GL: Ground Leaves

### Extraction of secondary metabolites

The secondary metabolites of all fungal isolates were extracted using ethyl acetate as solvent and the percent yield wascalculated. The percent yield of fungal extracts ranged from 5.2±0.22 to 27.51±1.02% as shown in Table 3. The fungalextract DOR-7 showed the highest percent yield 27.51±1.02%and DOR 6 showed the lowest percent yield 5.2±0.22%.

**Table 3.** Percent yield of fungal extracts.

Sample	Percent yield
DOR-1	12.37 ± 0.58
DOR-2	16.89 ± 0.74
DOR-3	6.42 ± 0.33
DOR-4	17.92 ± 0.53
DOR-5	13.12 ± 0.17
DOR-6	5.2 ± 0.22
DOR-7	27.51 ± 1.02
DOR-8	16.28 ± 0.88
DOR-9	16.41 ± 0.21
DOR-10	19.83 ± 0.67
DOR-11	10.02 ± 0.61
DOR-12	5.82 ± 0.19
DOR-13	11.72 ± 0.34

Values are means of three biological replicates (n =3).

### Molecular identification and phylogenetic analysis

**Figure 4.**
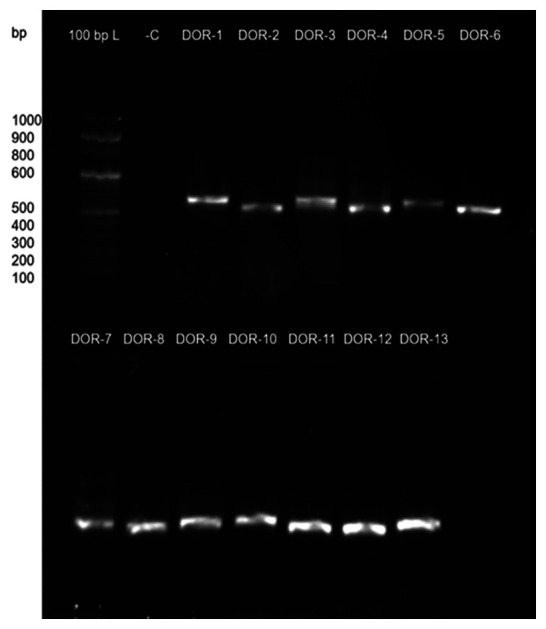
ITS gene PCR amplification of all fungal isolates (DOR-1 to DOR-13). 500–600bp sized bands observed after PCR amplification of the ITS gene of fungal samples D0R-1 to DOR-13.The PCR amplification products were analyzed by gel electrophoresis on 2% agarose (w/v) using 100bp ladder from Thermo-fisher. -C is negative control.

PCR amplification products of ITS regions together with the 5.8s rRNA gene (ITS 1-5.8S-ITS2 rDNA) were analyzed by gel electrophoresis on 2% agarose (w/v) as shown in Figure 4.

**Figure 5.**
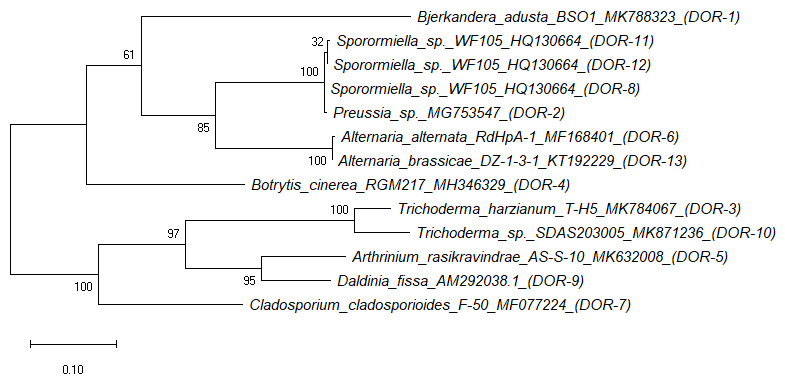
Phylogenetic tree generated from ITS-rDNA sequences. The tree with the highest log likelihood (-4362.92) is shown. Multiple sequence alignment of related species was performed using ClustalW. Phylogenetic analysis was carried out by Maximum Likelihood method using MEGA X software. Initial tree(s) for the heuristic search were obtained by Neighbor-Join and BioNJ algorithms to a matrix of pairwise distances estimated using the Maximum Composite Likelihood approach.

The fungal isolates DOR-1 to DOR-13 were subjected to molecular identification based on the ITS rDNA sequence analysis. The sequences are deposited in GenBank of NCBI with accession numbers MN696546 to MN696558. The details of putative taxonomic affinities are listed in Table 4. The phylogenetic tree of fungal isolates is shown in Figure 5. The 13 identified isolates were grouped into 9 genera: *Bjerkandera*, *Trichoderma, Preussia*, *Botrytis*, *Arthrinium*, *Alternaria*, *Cladosporium*, *Sporormiella* and *Daldinia. Bjerkandera adusta* belonged to phylum Basidiomycota while all others belonged to phylum Ascomycota. *Sporormiella* showed the highest colonization frequency accounting for 23% followed by *Alternaria* and *Trichoderma* with 15.4% each and the rest showed 7.7% each of colonization frequencies.

**Table 4.** Putative taxonomic affinities of fungal isolate.

Morphotype	Accession number	Closest relatives in NCBI	Reference sequence accession number	ITS identity (%)	Genus
DOR-1	MN696546	*Bjerkandera adusta*	MK788323	99%	*Bjerkandera*
DOR-2	MN696547	*Preussia* sp.	MG753547	100%	*Preussia*
DOR-3	MN696548	*Trichoderma harzianum*	MK784067	97.47%	*Trichoderma*
DOR-4	MN696550	*Botrytis cinerea*	MH346329	100%	*Botrytis*
DOR-5	MN696551	*Arthrinium rasikravindrae*	MK632008	100%	*Arthrinium*
DOR-6	MN696552	*Alternaria alternata*	MF168401	100%	*Alternaria*
DOR-7	MN696554	*Cladosporium* *cladosporioides*	MF077224	100%	*Cladosporium*
DOR-8	MN696555	*Sporormiella* sp.	HQ130664	100%	*Sporormiella*
DOR-9	MN696558	*Daldiniafissa sp.*	AM292038	100%	*Daldinia*
DOR-10	MN696549	*Trichoderma* sp.	MK871236	100%	*Trichoderma*
DOR-11	MN696556	*Sporormiella* sp.	HQ130664	100%	*Sporormiella*
DOR-12	MN696557	*Sporormiella* sp.	HQ130664	100%	*Sporormiella*
DOR-13	MN696553	*Alternaria brassicae*	KT192229	100%	*Alternaria*

### Detection of antioxidant activity using TLC

**Figure 6.**
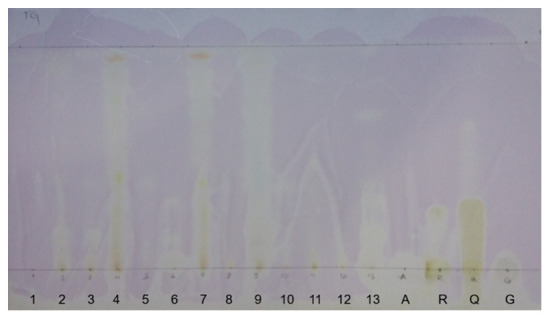
Qualitative analysis of antioxidant activity using thin layer chromatography (TLC). 20×20 alumina TLC plate showing yellow bands of all fungal extracts and standards: Ascorbic acid (A), Rutin (R), Quercitin (Q) and Gallic acid (G) after spraying with 10% AlCl _3_ solution followed by 0.04% DPPH solution.

Ascorbic acid, Rutin, Quercetin, and Gallic acid were used as standards. These standards produced yellowish bands on the TLC plate. 10 µl with 10 mg/ml concentration of fungal extracts were loaded to the plate. The extracts producing yellow bands on the purple background were considered as antioxidants. The purple background color was visualized after spraying the plate with 0.4%2,2-diphenyl-1-picrylhydrazy (DPPH) reagent. Of all the 13 extracts, five of them: DOR-4, DOR-6, DOR-7, DOR-9, and DOR-13 showed clearer yellow bands compared to other samples, hence indicating good DPPH radical scavenging activity ( Figure 6). The purple background was due to the presence of DPPH radical while the yellowish bands were formed due to the reduction of the DPPH radical. The yellowish bands indicate the presence of antioxidant compounds that can scavenge DPPH radical by donating an electron/hydrogen atom ^26^. The difference in the band intensities of developed yellow color could be due to the varied differences in the amount as well as the chemical characteristics of the compound present in of each extract.

### Determination of TPC

Phenols are basically secondary metabolites of plants or fungi formed against predation and protection. Nearly 8000 phenolics are known to exist, encompassing a wide range of molecules which can give up hydrogen atoms from their hydroxyl groups to radicals and form stable phenoxy radicals, hence playing an important role in antioxidant activity by reducing oxidative stress and the risks of cardiovascular diseases, osteoporosis as well as neurodegenerative diseases ^27^. Several methods for the determination of TPC have been introduced because of the heterogeneous nature of natural phenols. In this study, TPC was estimated using a modified Folin-Ciocalteu method.

**Figure 7.**
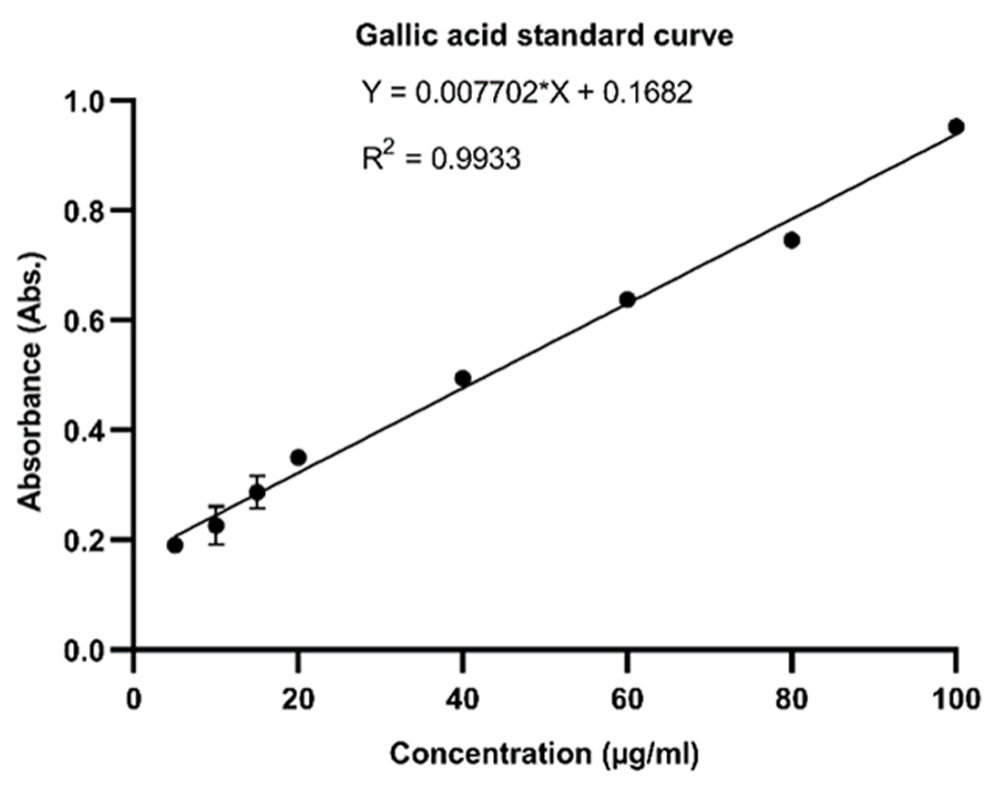
Calibration curve of Gallic acid. Regression equation of Gallic acid y=0.007702x+0.1682, R ^2^=0.9933 was obtained using the calibration curve. Error bars represent standard deviation.

**Figure 8.**
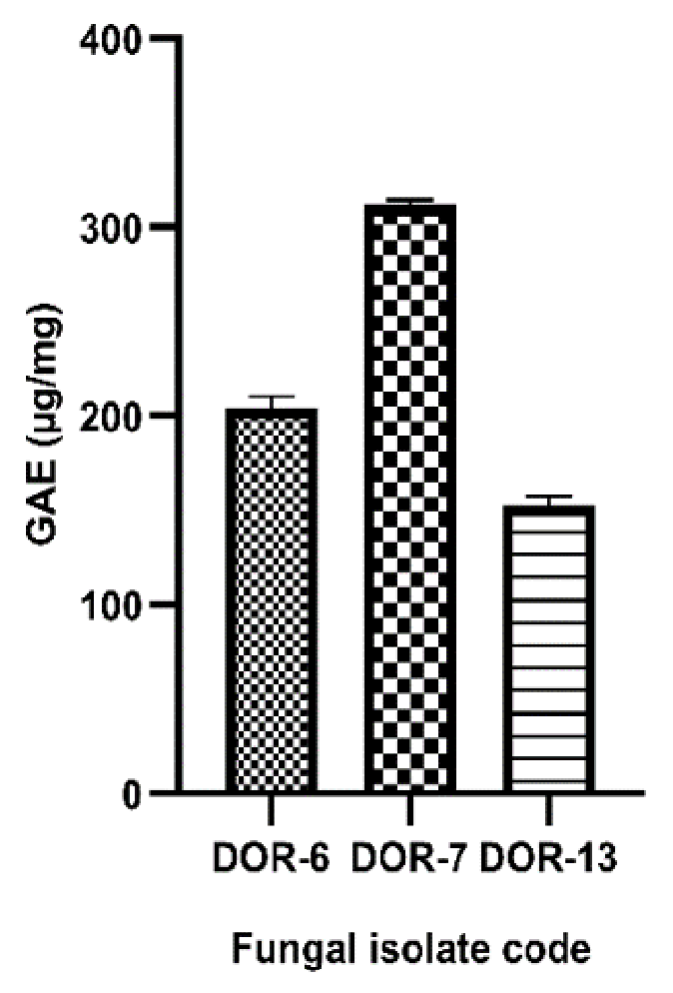
Total phenolic contents of endophytic fungal isolates DOR-6, DOR-7, and DOR-13. The phenolic content was calculated from the regression equation of the calibration curve (y=0.007702x+0.1682, R ^2^=0.9933), expressed in μg GAE per mg of dried extract. The samples DOR-6, DOR-7 and DOR-13 showed total phenolic contents of 204.1±6.144, 312.3±2.147, and 152.7±4.958 µg GAE/mg of dry extract respectively. The values are means of three biological replicates (n=3). Error bars in the graph represents standard deviation.

A blue-colored solution was observed due to the presence of phospomolybdic phosphotungstic-phenol *complex* when the fungal extracts reacted with the Folin-Ciocalteu reagent in alkaline medium. The phenolic content was calculated from the regression equation of the calibration curve (y=0.007702x+0.1682, R ^2^=0.9933), expressed in μg Gallic Acid Equivalent (GAE) per mg of dried extract. The total TPC of the samples showed large variations, with significant values of three samples: DOR-6, DOR-7 and DOR-13 with 204±6.144, 312.3±2.147 and 152.7±4.958 µg GAE/mg of dry extract, respectively. The calibration curve of Gallic acid and the GAE/mg value of fungal extracts showing high phenolic contents are shown in Figure 7 and Figure 8, respectively.

### Determination of TFC

Flavonoids include flavones, flavonols and condensed tannins that are secondary metabolites, whose antioxidant activity depends on the presence of free OH groups, especially 3-OH ^28^. Flavonoids are known to inhibit formation of reactive oxygen species, chelate trace elements involved in free-radical production, scavenge such reactive species and protect in antioxidant defenses ^29^. The flavonoids are reported to exhibit antioxidative, anticarcinogenic, anti-inflammatory, anti-aggregatory property and vasodilating effects ^30^. The TFC of the endophytic fungal extracts was estimated by NaNO _2 ^-^_Al(NO _3_) _3 ^-^_NaOH colorimetric method.

**Figure 9.**
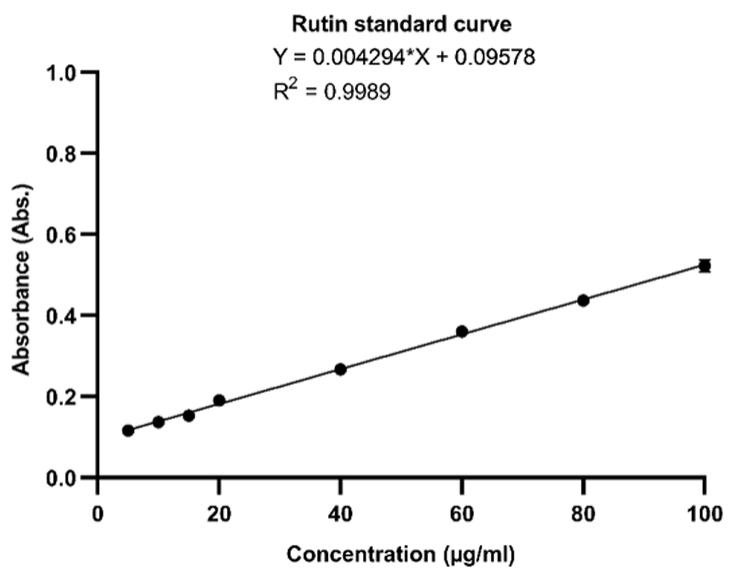
Calibration curve of Rutin. Regression equation y=0.004294x+0.09578, R ^2^=0.9989 of Rutin was obtained using the above calibration curve. Error bars represent standard deviation.

**Figure 10.**
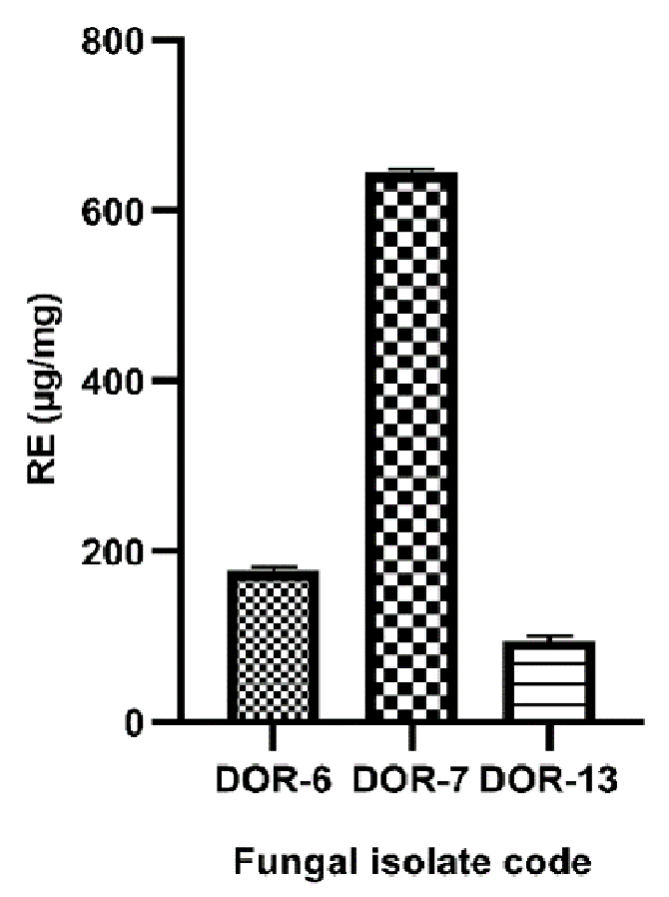
Total flavonoid contents of endophytic fungal isolates DOR-6, DOR-7, and DOR-13. The flavonoid content was calculated from the regression equation of the calibration curve (y=0.004294x+0.09578, R ^2^=0.9989), expressed in μg RE per mg of dried extract. The fungal extracts DOR-6, DOR-7 and DOR-13 showed total flavonoid contents of 177.9±2.911, 644.1±4.202, and 96.38±3.851 µg RE/mg dry extract respectively. The values are means of three biological replicates (n=3). Error bars in the graph represents standard deviation.

The flavonoid content was calculated from the regression equation of the calibration curve (y=0.004294x+0.09578, R ^2^=0.999), expressed in μg Rutin Equivalent (RE) per mg of dried extract. The TFC of the samples also showed large variations with significant values of three samples: DOR-6, DOR-7 and DOR-13 with values of 177.9±2.911, 644.1±4.202 and 96.38±3.851 µg RE/mg of dry extract, respectively. The calibration curve of Rutin and the RE/mg value of fungal extracts showing high flavonoid contents are shown in Figure 9 and Figure 10, respectively.

### DPPH radical scavenging activity

DPPH assay is a widely used method to evaluate the antioxidant ability of extracts. It measures the ability of compounds to act as hydrogen donors or free radical scavengers ^31, 32^. Various types of antioxidant compounds may be produced by fungal endophytes including multiple flavonoid compounds, saponin, phenols, sterols, terpenoids, etc., which may remove DPPH free radicals ^26^. These antioxidant compounds have important health effects such as reducing the risk of cancer, heart disease, and neuro-degenerative disorders that are found in abundant amounts in plants, vegetables, fruits, and natural products ^33^. DPPH is a stable nitrogen-centered free radical that can readily undergo scavenging by antioxidants ^34^. DPPH gives rise to the deep violet color, with maximum absorption at a wavelength of 517 nm. DPPH reduces to diphenyl picrylhydrazine after the odd electron of a nitrogen atom in DPPH receives an electron or hydrogen atom from antioxidants and changes its color from the deep violet to pale yellow ^35^. As antioxidant donates a proton to this radical, the absorbance decreases due to the disappearance of DPPH radical.

**Figure 11.**
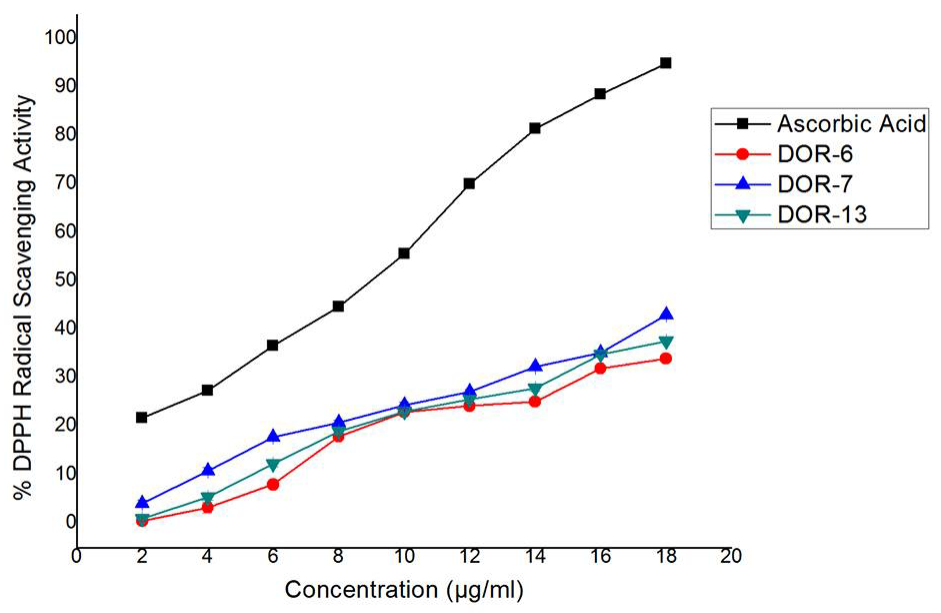
DPPH radical scavenging activity of ascorbic acid and fungal extracts DOR-6, DOR-7 and DOR-13. Comparison of % DPPH radical scavenging activity of selected fungal extracts DOR-6, DOR-7, and DOR-13 with the standard antioxidant Ascorbic acid at different concentrations (2, 4, 6, 8, 10, 12, 14, 16, and 18 µg/ml). The radical scavenging activity of the standard (Ascorbic acid), as well as the EA fungal extracts, were calculated using the formula: DPPH radical scavenging activity (%) = (Abs. control – Abs. sample)/Abs. control x 100, where Abs. control is the absorbance of DPPH radical solution without samples.

**Figure 12.**
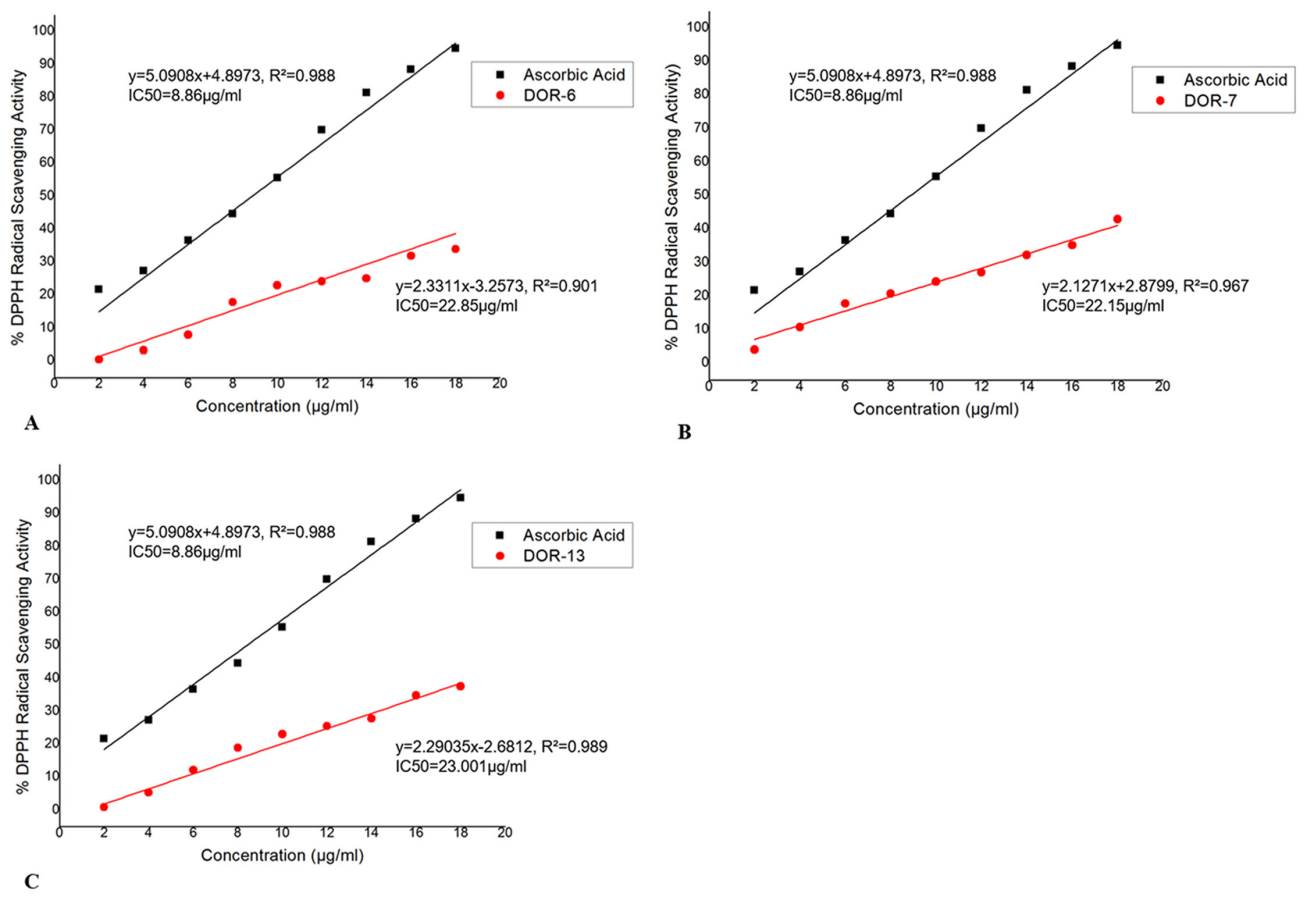
Determination and comparison of IC _50_ of fungal extracts DOR-6, DOR-7 and DOR-13 with standard Ascorbic acid. ( **A**) Comparison of 50% inhibition concentration of fungal extract DOR-6 with the standard antioxidant Ascorbic acid. The IC _50_ value of standard ascorbic acid, fungal extract DOR-6 was determined from equations y=5.0908x+4.8973, y=2.3311x–3.2573 respectively. ( **B**) Comparison of 50% inhibition concentration of fungal extract DOR-7 with the standard antioxidant Ascorbic acid. The IC _50_ value of standard ascorbic acid, fungal extract DOR-7 was determined from the equations y=5.0908x+4.8973, y=2.1271x+2.8799 respectively. ( **C**) Comparison of 50% inhibition concentration of fungal extract DOR-7 with the standard antioxidant Ascorbic acid. The IC _50_ value of standard ascorbic acid, fungal extract DOR-7 was determined from the equations y=5.0908x+4.8973, y=2.29035x–2.6812 respectively.

Among the 13 fungal extracts, three of the extracts, DOR-6, DOR-7, and DOR-13, showed the highest radical scavenging activity. The radical scavenging activity of Ascorbic acid as well as the fungal extracts DOR-6, DOR-7 and DOR-13 were found to be dose dependent ( Table 5 and Figure 11). IC _50_ values of these fungal extracts and ascorbic acid was calculated from the equation obtained from linear fitting curve as shown in Figure 12. The fungal extracts DOR-6, DOR-7 and DOR-13 showed a significant radical scavenging activity with a 50% inhibition (IC _50_) at a concentration of 22.85, 22.15 and 23.001 µg/ml as compared with standard ascorbic acid that showed 50% inhibition at a concentration of 8.86 µg/ml.

**Table 5.** Percentage of radical scavenging activity of Ascorbic acid and fungal extracts DOR-6, DOR-7 and DOR-13 at different concentrations.

Concentration	Percentage radical scavenging activity			
	Ascorbic Acid	DOR-6	DOR-7	DOR-13
2	21.76±0.54	0.5±0.15	4.09±0.67	0.96±0.27
4	27.36±0.58	3.28±1.05	10.83±0.49	5.37±0.59
6	36.70±1	8.01±0.13	17.85±0.3	12.25±0.53
8	44.67±0.11	17.89±0.09	20.81±0.49	18.94±0.57
10	55.64±0.65	22.99±0.46	24.41±0.56	23.09±0.54
12	70.13±0.33	24.22±0.28	27.14±0.54	25.54±0.51
14	81.51±0.5	25.14±0.34	32.33±0.13	27.86±0.61
16	88.57±0.29	31.96±0.65	35.24±0.18	34.88±0.32
18	94.89±0.22	34.01±0.39	43.03±0.18	37.61±0.24

Values are means of three biological replicates (n =3).

### Determination of antimicrobial activity

Endophytic fungi have the ability to produce a large variety of bioactive compounds that can protect the plants against various pathogens ^7, 36^. The screening of all the fungal extracts was conducted using agar well diffusion method. 20 mg/ml concentration of the sample was used. Gentamycin was used as positive control and 10% DMSO was used as a negative control. Among the 13 fungal extracts three of the extracts: DOR-6, DOR-7, and DOR-13 showed the highest antimicrobial activity during primary screening against *Escherichia coli* (ATCC 25922) and *Staphylococcus aureus (*ATCC 12600) and were selected for secondary screening.

**Figure 13.**
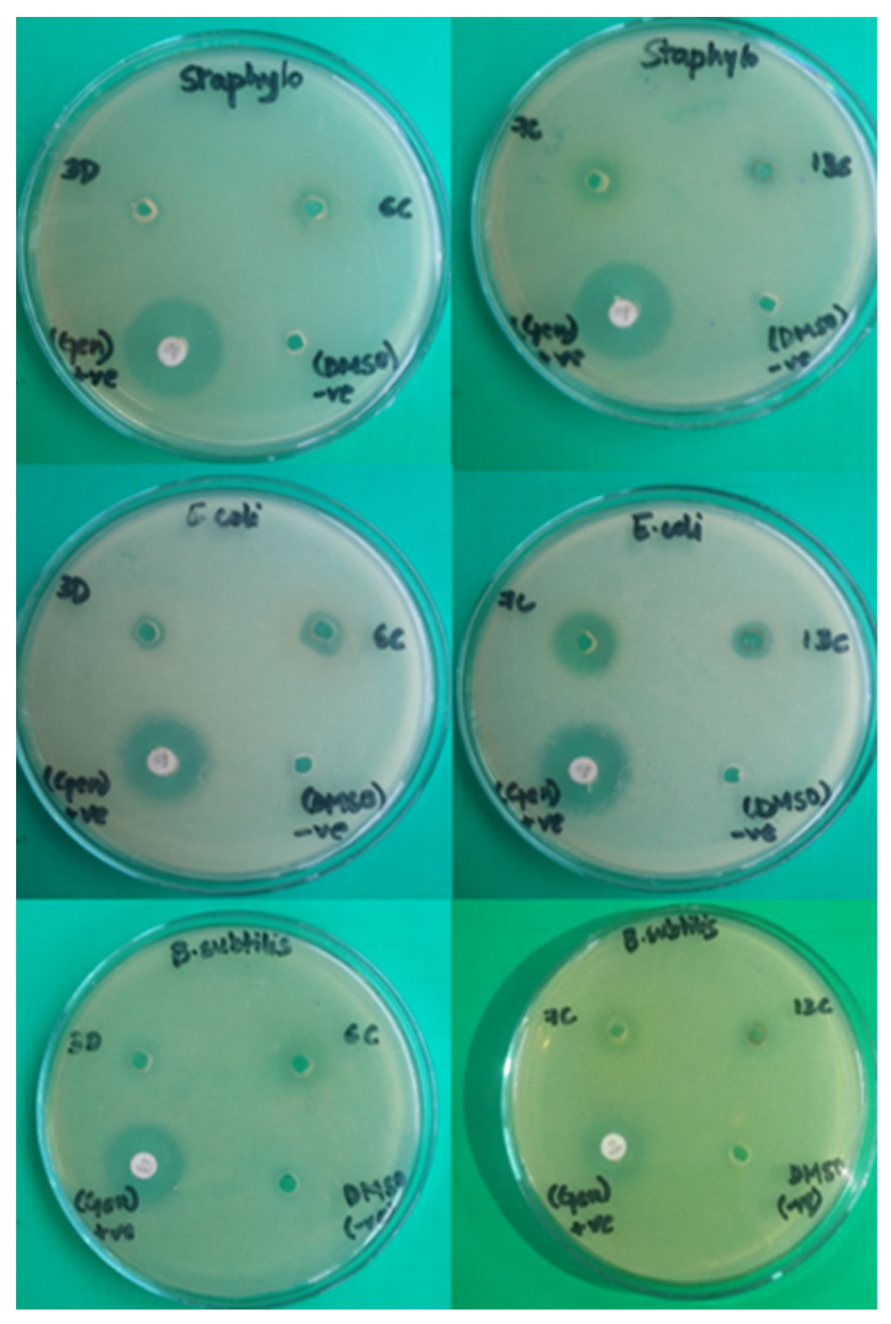
Antimicrobial activity of fungal extracts DOR-6, DOR-7 and DOR-13. Antimicrobial activity of fungal extracts DOR-6, DOR-7 and DOR-13 was determined using a gram-negative bacterial strain: *Escherichia coli* (ATCC 25922), and two gram-positive bacterial strains: *Staphylococcus aureus* (ATCC 12600) and *Bacillus subtilis* (ATCC6633) with 10 µg Gentamycin disc, as a positive control and DMSO as negative control.

**Figure 14.**
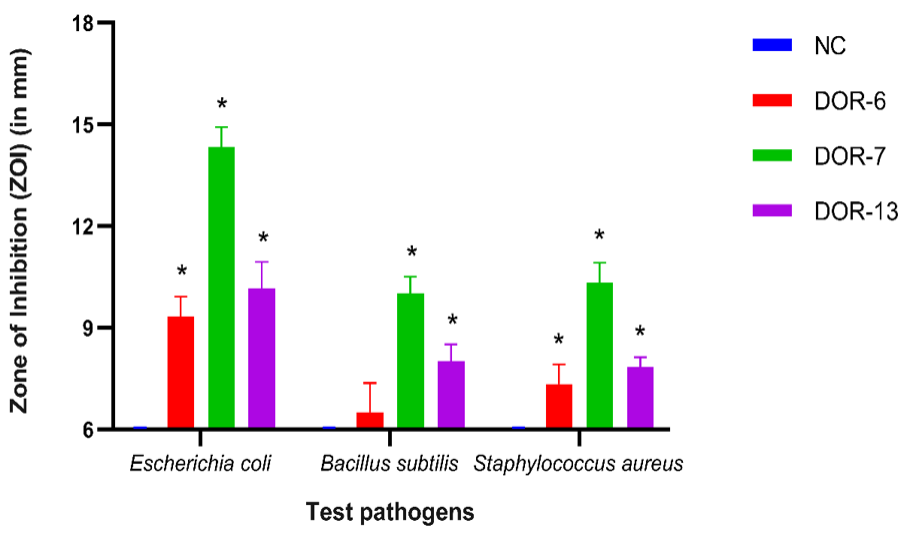
Comparison of antimicrobial activity of fungal extracts DOR-6, DOR-7 and DOR-13 with negative control. Comparison of mean zones of inhibition measurements (mm) of fungal crude extracts (DOR-6, DOR-7, and DOR-13) with DMSO as a negative control (NC) for bacterial test pathogens. Error bars represent standard deviation. An asterisk (*) indicates a significant difference in mean zones of inhibition between fungal crude extracts and negative control observed for each test pathogen (compared using Dunnett’s test, P < 0.05). If no inhibition was seen, a value of 6 was assigned, which was the diameter of the agar-well used.

**Figure 15.**
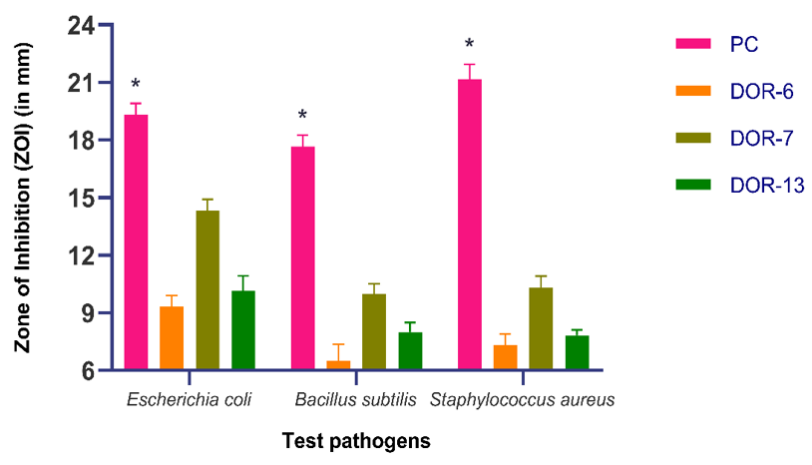
Comparison of antimicrobial activity of fungal extracts DOR-6, DOR-7 and DOR-13 with positive control. Comparison of mean zones of inhibition measurements (mm) of fungal crude extracts (DOR-6, DOR-7 and DOR-13) with antibiotic - 10 µg Gentamycin disc, as a positive control (PC) for bacterial test pathogens. Error bars represent standard deviation. An asterisk indicates significant difference in mean zones of inhibition between positive control and fungal crude extracts observed for each test pathogen (compared using Dunnett’s test, P < 0.05). The less potency of the fungal extract in comparison to antibiotic disc of Gentamycin can be attributed to the crude nature of the extracts used. If no inhibition was seen, a value of 6 was assigned, which was the diameter of the agar-well used.

During the secondary screening, the antimicrobial activity of these three fungal extracts was appraised on three bacterial strains: Gram-positive bacteria *- Staphylococcus aureus (*ATCC 12600), and *Bacillus subtilis (*ATCC 6633), and a Gram-negative bacterium: *Escherichia coli (*ATCC 25922) ( Figure 13). DOR-6 showed inhibition diameter of 9.333±0.577, 7.33±0.557 and 6.5±.866 mm, DOR-7 showed inhibition diameter of 14.33±0.577, 10.33±0.577 and 10±0.5 mm, and DOR-13 showed inhibition diameter of 10.167±0.764, 8±0 and 8±0.5 mm against *E. coli*, *S. aureus* and *B. subtilis,* respectively ( Table 6). MIC value of these three extracts was further assessed. Positive control Gentamycin showed 19.333±0.577, 21.167±0.764 and 17.667±0.557 mm inhibition diameter against *E. coli*, *S. aureus* and *B. subtilis*. DOR-7 and DOR-13 showed good antimicrobial activity, hence were selected for determination of MIC. The figure exhibiting the comparison of zone of inhibition of fungal extracts DOR-6, DOR-7 and DOR-13 with negative and positive control is shown in Figure 14 and Figure 15, respectively.

**Table 6.** Zones of inhibition of fungal crude extracts DOR-6, DOR-7 and DOR-13 against bacterial test pathogens *E.coli, S*. aureus and *B. subtilis*.

Sample	Inhibition diameter (mm)		
	*Escherichia coli*	*Staphylococcus aureus*	*Bacillus subtilis*
DOR-6	9.33±0.58	7.33±0.58	6.5±0.87
DOR-7	14.33±0.57	10.33±0.58	10±0.5
DOR-13	10.17±0.76	8±0	8±0.5

Values are expressed as Mean±SD.

### Determination of MIC

Crude extracts showing potent antimicrobial activity was further examined for MIC by tube dilution technique. The maximum inhibition diameter was shown by fungal extracts DOR-7 and DOR- 13 in *E. coli.* So, the minimum inhibitory concentration of these two extracts was assessed using *E. coli* as an indicator organism. Kanamycin sulphate was used as positive control.

**Figure 16.**
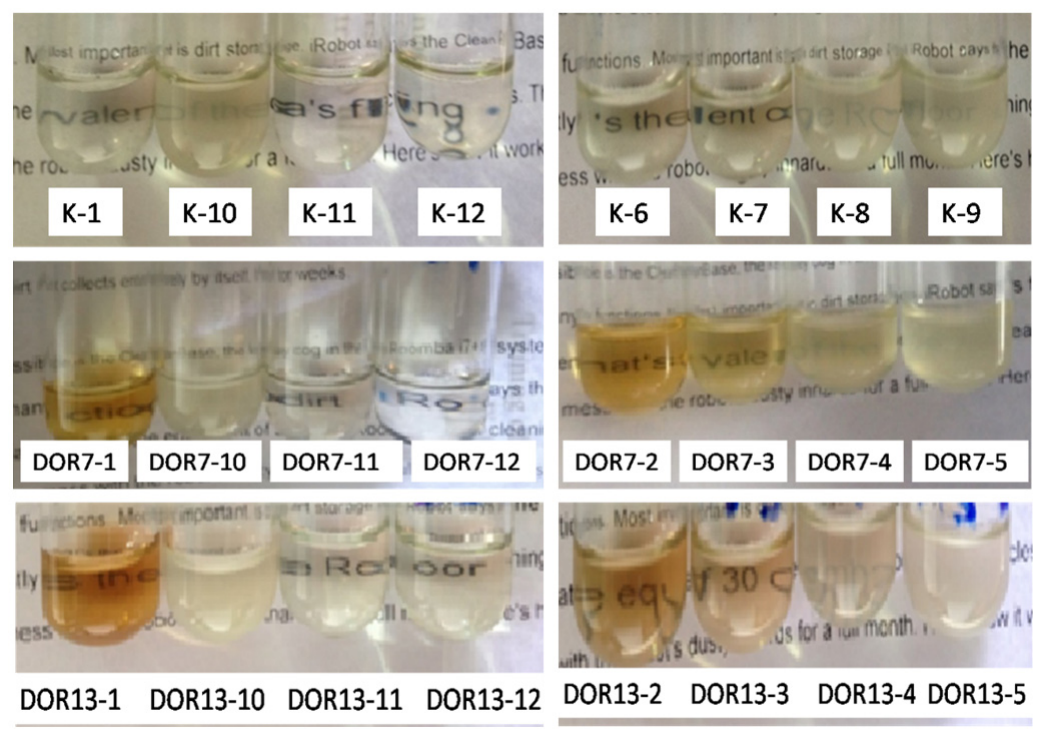
Minimum inhibitory concentration (MIC) of kanamycin sulfate and fungal extracts DOR-7 and DOR-13. MIC of kanamycin sulfate was observed at K-7 i.e. at 15.625 µg/ml concentration. MIC of extract DOR-7 and DOR-13 observed at DOR7-3 (250 µg/ml) and DOR13-3 (250 µg/ml) respectively. 11 and 12 are negative controls and 1 and 10 represent the highest and lowest concentration, i.e. 1 mg/ml and 1.9531 µg/ml, respectively.

The microorganisms were subjected to a range of antibiotic concentrations: 1000, 500, 250, 125, 62.5, 31.25, 15.625, 7.8125, 3.9062, and 1.9531 µg/ml. Serial dilution of the fungal extracts was performed in the same concentration range between each successive test tube. The macroscopic inhibition of growth was measured by observing the absence of turbidity in the medium ( Figure 16). The MIC of samples DOR-7, as well as DOR-13, was determined to be 250 µg/ml. It means the concentration of a sample less than 250 µg/ml showed growth of bacteria. The MIC of kanamycin sulphate was 15.625 µg/ml, i.e. bacterial growth was observed only in those test tubes containing antibiotic concentration less than 15.625 µg/ml. The results of the present study suggest that 250 µg/ml of fungal extracts DOR-7 and DOR-13 consists of bioactive compounds equivalent to 15.625 µg/ml kanamycin sulphate. These bioactive compounds with antibacterial property might be the potential source for the development of antimicrobial compounds.

### Determination of cytotoxicity by brine shrimp assay

**Figure 17.**
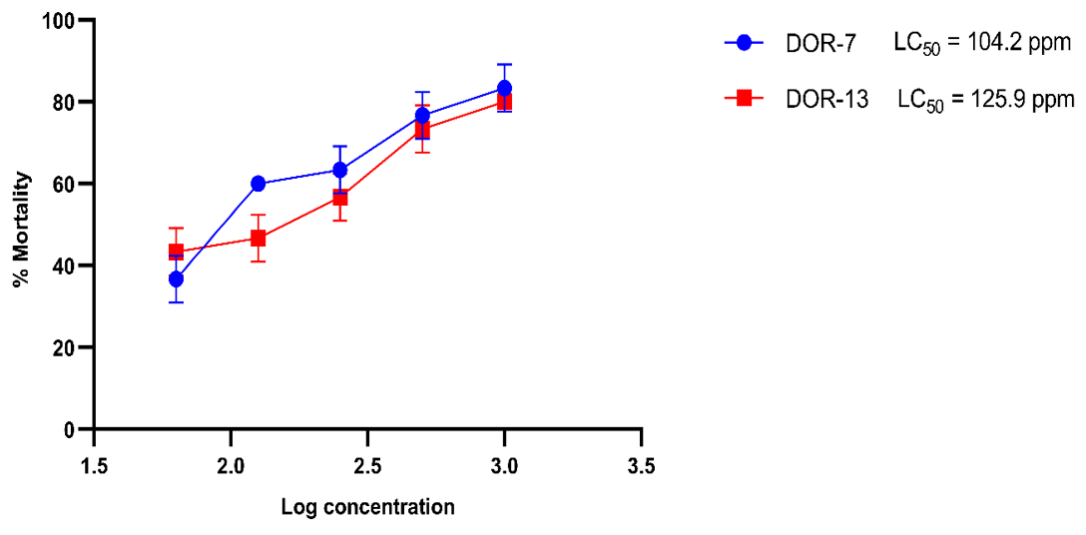
Log of concentration vs percentage mortality of fungal extracts DOR-7 and DOR-13. The cytotoxicity of the fungal extracts at different concentrations: 1,000, 500, 250, 125, and 62.5 ppm was determined by their ability to kill the shrimp nauplii in 24 hours. The lethal concentration required to kill half the population (LC _50_) was calculated. 1% doxorubicin and 1% DMSO were used as positive and negative control respectively.

Brine shrimp ( *Artemia salina* Leach) is an invertebrate inhabiting saline aquatic and marine ecosystem. It can be used in a laboratory bioassay in order to determine the toxicity of plants by the estimation of the medium lethality concentration LC _50_^37^. In this study, cytotoxicity test was done by brine shrimp bioassay method. We calculated the cytotoxicity of our fungal extracts by their determining their ability to kill the shrimp nauplii in 24 hours. The lethal concentration required to kill half the population (LC _50_) was calculated. 1% doxorubicin and 1% DMSO were used as positive and negative control respectively. Death percentage of nauplii in 24 hours was calculated and is shown in Table 7. A graph of a log of extract concentration in ppm versus death percentage was plotted ( Figure 17).

**Table 7.** Brine shrimp bioassay for fungal extracts DOR-7 and DOR-13 showing death percentage of nauplii after 24 hours.

Sample	Conc (A) (ppm)	Log of A	Initial Nauplii	Nauplii survival (n=1)	Nauplii survival (n=2)	Nauplii survival (n=3)	% Mortality (n=1)	% Mortality (n=2)	% Mortality (n=3)	LC _50_ (ppm)
**DOR-7**	1000	3	10	2	2	1	80	80	90	104.2
	500	2.7	10	3	2	2	70	80	80	
	250	2.4	10	3	4	4	70	60	60	
	125	2.1	10	4	4	4	60	60	60	
	62.5	1.8	10	6	6	7	40	40	30	
**DOR-13**	1000	3	10	2	2	2	80	80	80	125.9
	500	2.7	10	2	3	3	80	70	70	
	250	2.4	10	4	5	4	60	50	60	
	125	2.1	10	5	5	6	50	50	40	
	62.5	1.8	10	6	5	6	40	50	40	

LC _50_ of extracts DOR-7 and DOR-13 were 104.2 ppm and 125.9 ppm, respectively. In our study, both extracts showed a low value of LC _50_ (<200 µg/ml), which is an indication of the presence of the potent cytotoxic compound. Throughout our experiment, the shrimp nauplii did not receive food; therefore the death of the nauplii can be considered either due to the effect of our bioactive fungal extract or because of starvation. However, any hatched nauplii still feed on their yolk-sac, they can survive for up to 48 hours without food ^38^. In addition, we had also used a control sample containing only nauplii for ensuring the mortality effect of our fungal extract. Since brine shrimp lethality assay is a primary assay to detect cytotoxic property, further experiments are required to establish the cytotoxicity of the fungal extracts against cell lines.

## Conclusions

Endophytic fungi are largely underexplored in the discovery of natural products. To date, only a few plants are investigated for their endophytic biodiversity and their potential to produce bioactive secondary metabolites. Nepal has an abundance of medicinal plants, so this research helps shed a light in the possibilities of discovering novel compounds with pharmaceutical potential from a variety of endophytic fungi residing within them. Our research primarily concludes Himalayan yew to be a promising reservoir for large endophytic diversity with the capability to produce natural compounds showing significant antioxidant, antimicrobial and cytotoxic properties. A total of 9 different genera of fungi were isolated from 16 *Taxus* samples, which further suggests that this diversity could offer an abundant source of varied novel metabolites. The fungal extracts isolated from three endophytic fungi, *Alternaria alternata*, *Cladosporium cladosporioides* and *Alternaria brassicae,* showed significant bioactivity. Alternaria sp. have also exhibited a myriad of biological functions such as phytotoxic, cytotoxic, antimicrobial and chemotherapeutic agent like porritoxin ^39– 41^. Similarly, several studies conducted on C. cladosporoids have shown bioactive compounds like p- methylbenzoic acid, Ergosterol peroxide and Calphostin as well as enzymes like pectin methylesterase, polygalacturonase and chlorpyrifos hydrolase ^42- 45^. Likewise, these crude fungal extracts upon purification and further identification of the bioactive compounds can be a fascinating source for novel pharmaceutical agents. Although these compounds are produced in small quantities in nature, these promising endophytes can be further employed to fast growth and amplification through isolation, genetic manipulation, and industrial scale-up on an industrial level. Moreover, the utilization of endophytes as alternative sources of bioactive compounds also eliminates the exploitation of the host plant, hence conserving biodiversity.

## Data availability

### Underlying data

Sequences of fungal isolates (DOR-1 to DOR-13) are deposited in GenBank of NCBI with accession numbers MN696546 to MN696558. See Table 4 for a full list of accession numbers.

Open Science Framework: Evaluation of antimicrobial, antioxidant and cytotoxic properties of bioactive compounds produced from endophytic fungi of Himalayan Yew ( *Taxus wallichiana*) in Nepal, https://doi.org/10.17605/OSF.IO/73PAX ^46^.

This project contains the following underlying data:

- Uncropped, unedited images for Figure 4 and Figure 6.
- DPPH, TPC, TFC results
- Agar well diffusion (Primary and secondary screening results)
- Brine shrimp assay results
- Percent yield results

Data are available under the terms of the Creative Commons Zero "No rights reserved" data waiver (CC0 1.0 Public domain dedication).
