# Supplementary material for: Evaluation of antimicrobial, antioxidant and cytotoxic properties of bioactive compounds produced from endophytic fungi of Himalayan yew ( Taxus wallichiana) in Nepal
Source: F1000Res. 2020 Oct 5;9:379. Originally published 2020 May 19. [Version 2] doi: 10.12688/f1000research.23250.2 (PMC7551515; doi:10.12688/f1000research.23250.2)
Supplement: Peer review history

# Peer review: Reviewer response for version 2

**DOI:** 10.5256/f1000research.29866.r72442

**Published:** 2020-10-12

**Authors:** Dipesh Dhakal

**Affiliations:** 1 Department of Medicinal Chemistry, University of Florida, Gainesville, FL, USA

The current version of the manuscript is refined and interesting for wide readership.

 In addition, the authors have either duly rectified the contents or provided appropriate responses to the reviewer's comments. Hence, the current version of manuscript can be accepted.

Is the work clearly and accurately presented and does it cite the current literature?

Yes

If applicable, is the statistical analysis and its interpretation appropriate?

Partly

Are all the source data underlying the results available to ensure full reproducibility?

Yes

Is the study design appropriate and is the work technically sound?

Partly

Are the conclusions drawn adequately supported by the results?

Yes

Are sufficient details of methods and analysis provided to allow replication by others?

Yes

Reviewer Expertise:

Natural Product Biosynthesis, Bioactivity of natural products as antibacterial, anticancer, anti-oxidant etc, Engineering of natural products.

I confirm that I have read this submission and believe that I have an appropriate level of expertise to confirm that it is of an acceptable scientific standard.

# Peer review: Reviewer response for version 2

**DOI:** 10.5256/f1000research.29866.r72443

**Published:** 2020-10-06

**Authors:** Anaya Pokhrel

**Affiliations:** 1 University of Minnesota, Saint Paul, MN, USA

The research article looks good after revision. I am happy with the revised article and I do not have any further comments to make.

Is the work clearly and accurately presented and does it cite the current literature?

Yes

If applicable, is the statistical analysis and its interpretation appropriate?

Yes

Are all the source data underlying the results available to ensure full reproducibility?

Yes

Is the study design appropriate and is the work technically sound?

Yes

Are the conclusions drawn adequately supported by the results?

Yes

Are sufficient details of methods and analysis provided to allow replication by others?

Partly

Reviewer Expertise:

Microbiology, molecular biology, and natural products.

I confirm that I have read this submission and believe that I have an appropriate level of expertise to confirm that it is of an acceptable scientific standard.

# Peer review: Reviewer response for version 1

**DOI:** 10.5256/f1000research.25669.r70764

**Published:** 2020-09-07

**Authors:** Dipesh Dhakal

**Affiliations:** 1 Department of Medicinal Chemistry, University of Florida, Gainesville, FL, USA

The manuscript provides showcase about the diversified fungal consortium existing in Taxus wallichiana. Further they have isolated secondary metabolites and analyzed their antioxidant, antimicrobial and anticancer activities.The findings are very interesting to cater the biological importance of such potent fungus. However, the study has room for improvement.

1. The author can analyze the secondary metabolite profile by HPLC and GC, to determine whether they are already known molecule or entirely new molecules.
2. The author can perform MTT assay against few cancer cell lines and normal cell line, which will provide better insight on practical application of such metabolites.
3. The author showed that the extract has better activity against Gram negative strains, hence it would be interesting if author would add additional Gram negative bacterias as Pseudomonas or ESKAPE pathogens for their testing.
4. Does the author isolate entirely new species of fungus or they recovered already known species of fungus. In case the fungus are known, then the author can include some information about potential compounds from such fungal species.
5. In section "Determination of MIC", please correct 1000 instead of 1. 
6. In section, Extraction of secondary metabolites, the author should mentioned that they weighed the extract hence the author should provide information about the total yield.
7. I find the author have reiterated the datas on figures as well as table, hence they can just summarize in Figure and include the data in figure legend for e.g. Table 4 and Figure 8 can be integrated.

Is the work clearly and accurately presented and does it cite the current literature?

Yes

If applicable, is the statistical analysis and its interpretation appropriate?

Partly

Are all the source data underlying the results available to ensure full reproducibility?

Yes

Is the study design appropriate and is the work technically sound?

Partly

Are the conclusions drawn adequately supported by the results?

Yes

Are sufficient details of methods and analysis provided to allow replication by others?

Yes

Reviewer Expertise:

Natural Product Biosynthesis, Bioactivity of natural products as antibacterial, anticancer, anti-oxidant etc, Engineering of natural products.

I confirm that I have read this submission and believe that I have an appropriate level of expertise to confirm that it is of an acceptable scientific standard, however I have significant reservations, as outlined above.

# Response

**Published:** 2020-09-24

**Authors:** Dhurva P. Gauchan

**Affiliations:** Kathmandu University, Nepal

Dear Dr. Dipesh Dhakal,

 Thank you for your comments. Your comments helped us to add a few points to make the manuscript clearer. We addressed all the issues in version 2 of the manuscript as suggested.

 The suggestion for the employment of HPLC/GC for identification of isolated bioactive compounds, MTT assay, and antimicrobial assay on other gram-negative bacteria on our research has been acknowledged and research being currently investigated adopting various parameters. Matter of fact, our next phase of the research focuses on the identification of bioactive compounds using HPLC/LCMS and its effect on various cancer cell lines doing MTT assay. At the advance level, we have planned to evaluate the effectiveness of bioactive compounds extending our experiments on the animal models which will help to develop anticancer drugs in near future. We recovered already identified species of fungi. Therefore, some information about bioactive compounds previously isolated and studied from the identified fungal species is added to the conclusion section as suggested.

 In the determination of the MIC section, the initial diluted concentration is changed from 1 to 1000 μg/ml. Thank you for pointing out the error.

 The percent yield of fungal extracts is added in Table 3. The formula used for calculation of the yield is also added in the method section.

 Table 4 and Figure 8; Table 5 and Figure 10 are integrated as advised.

 Regards

 Dhurva P. Gauchan

# Peer review: Reviewer response for version 1

**DOI:** 10.5256/f1000research.25669.r63720

**Published:** 2020-07-02

**Authors:** Anaya Pokhrel

**Affiliations:** 1 University of Minnesota, Saint Paul, MN, USA

The manuscript entitled “Evaluation of antimicrobial, antioxidant and cytotoxic properties of bioactive compounds produced from endophytic fungi of Himalayan yew ( *Taxus wallichiana*) in Nepal” discusses isolation of endophytic fungi. Antimicrobial, antioxidant, and cytotoxic properties of bioactive compounds isolated from endophytic fungi is studied. The manuscript is clear, concise, and flows smoothly. However, there are minor corrections suggested before consideration of the manuscript for indexing.

Suggestions

**Introduction section**

 Sentence:

 Some research indicates various medicinal properties of *T. wallichiana, *such as analgesic, antipyretic, anti-inflammatory, immunomodulatory, antibacterial, antifungal, antiplatelet, antispasmodic activities antiallergic, anticonvulsant, antiosteoporotic, , and vasorelaxing effect ^3^^–^^6^.

 Antispasmodic activities: remove activities in this sentence

 antiosteoporotic, ,: Remove one comma

**Method section**

 Under the title “Molecular screening and phylogenetic analysis” in this sentence:

- The obtained pure DNA was run on 0.8% agarose gel electrophoresis and the bands were observed

Suggestions:

- “Pure DNA” is a claim not supported by evidence, please replace it with genomic DNA instead.
- Replace the word “run” with “analyzed using” and remove the part that says the bands were observed. This description belongs to the result section.
- In the PCR amplification condition, please add the number of cycles to make the data reproducible.
- Units for volume and time are not consistent. There is space right after the number in some cases while there is no space in some other. Please make it consistent.

For example in this section:

- PCR was performed using all reagents from Thermofisher, in a 25μl reaction mixture using 5 μl template DNA, 0.25 μl of 1.25 U Taq DNA polymerase, 2.5 μl of 10X Amplification buffer, 0.8μl of 10mM dNTP, 0.75 μl of 50mM MgCl2, 13.2 μl PCR grade water, 1.25μl of 10μM each of forward and reverse primer. PCR amplification was carried out in BioRad300 thermocycler. The thermo-cycling conditions involved an initial denaturation at 94°C for 3 mins, 37 cycles of 30 secs at 94°C, 30 secs at 55°C, 1 min at 72°C and a final extension at 72°C for 5 mins. The PCR amplification products were analyzed by gel electrophoresis on 2% agarose (w/v).
- Initial tree(s) for the heuristic search were obtained by Neighbor-Join and BioNJ algorithms to a matrix of pairwise distances estimated using the Maximum Composite Likelihood (MCL) approach, and then selecting the topology with superior log likelihood value ^19^. 

**Result section**

 In the following sentence:

*Sporormiella *showed the highest colonization frequency accounting for 23% followed by *Alternaria *and *Trichoderma *with 15.4% each and the rest showed 7.7% each of colonization frequencies.

 The colonization frequencies are discussed. However, the colonization frequency is not discussed in the method section. Please describe the method used for the calculation of colonization frequency somewhere in the methods section.

Is the work clearly and accurately presented and does it cite the current literature?

Yes

If applicable, is the statistical analysis and its interpretation appropriate?

Yes

Are all the source data underlying the results available to ensure full reproducibility?

Yes

Is the study design appropriate and is the work technically sound?

Yes

Are the conclusions drawn adequately supported by the results?

Yes

Are sufficient details of methods and analysis provided to allow replication by others?

Partly

Reviewer Expertise:

Microbiology, molecular biology, and natural products.

I confirm that I have read this submission and believe that I have an appropriate level of expertise to confirm that it is of an acceptable scientific standard, however I have significant reservations, as outlined above.

# Response

**Published:** 2020-09-24

**Authors:** Dhurva P. Gauchan

**Affiliations:** Kathmandu University, Nepal

Dear Dr. Anaya Pokhrel,

 Thank you for your meticulous observation especially in the materials and methods section which will definitely improve the quality of the manuscript. We addressed all the issues in version 2 of the manuscript as suggested.

 All typographical corrections suggested in the introduction and method sections have been corrected accordingly.

 The number of PCR cycles was included in the original version. (The thermo-cycling conditions involved an initial denaturation at 94°C for 3 mins, 37 cycles of 30 secs at 94°C, 30 secs at 55°C, 1 min at 72°C and a final extension at 72°C for 5 mins.) The units and spacing in between are made consistent throughout the paper as suggested.

 We have also added the method for calculation of colonization frequency in the revised paper.

 Regards

 Dhurva Prasad Gauchan
